# Supplementary material for: Integrating genomic structural equation modeling and experimental validation to unravel the genetic basis of male genital lichen sclerosus
Source: Front Immunol. 2026 Jul 16;17:1857945. doi: 10.3389/fimmu.2026.1857945 (PMC13435250; doi:10.3389/fimmu.2026.1857945)
Supplement: Supplementary file 6 [file SupplementaryFile5.zip › result/fuma_PSYCH_factor_sCCA1_focus.chr3.chr3.42540002.45165153.pop1.pdf]

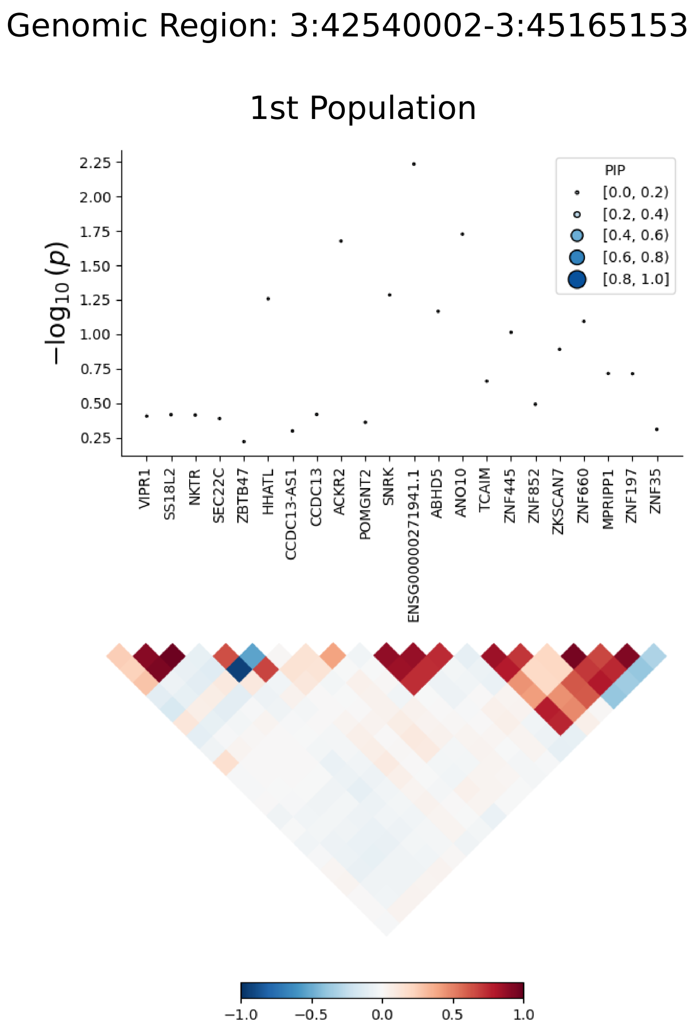

Genomic Region: 3:42540002-3:45165153
1st Population
$-\log_{10}(p)$
2.25
2.00
1.75
1.50
1.25
1.00
0.75
0.50
0.25
PIP
[0.0, 0.2)
[0.2, 0.4)
[0.4, 0.6)
[0.6, 0.8)
[0.8, 1.0]
VIPR1
SS18L2
NKTR
SEC22C
ZBTB47
HHATL
CCDC13-AS1
CCDC13
ACKR2
POMGNT2
SNRK
ENSG00000271941.1
ABHD5
ANO10
TCAIM
ZNF445
ZNF852
ZKSCAN7
ZNF660
MPRIPP1
ZNF197
ZNF35
−1.0
−0.5
0.0
0.5
1.0
